# Augmenter of Liver Regeneration Reduces Ischemia Reperfusion Injury by Less Chemokine Expression, Gr-1 Infiltration and Oxidative Stress

**DOI:** 10.3390/cells8111421

**Published:** 2019-11-12

**Authors:** Thomas S. Weiss, Madeleine Lupke, Rania Dayoub, Edward K. Geissler, Hans J. Schlitt, Michael Melter, Elke Eggenhofer

**Affiliations:** 1 University Children Hospital (KUNO), University Hospital Regensburg, 93053 Regensburg, Germany; madeleine@lupke.net (M.L.); Rania.Dayoub@ukr.de (R.D.); Michael.melter@ukr.de (M.M.); 2 Center for Liver Cell Research, University Hospital Regensburg, 93053 Regensburg, Germany; 3 Department of Surgery, University Hospital Regensburg, 93053 Regensburg, Germany; Edward.geissler@ukr.de (E.K.G.); Hans.schlitt@ukr.de (H.J.S.); Elke.eggenhofer@ukr.de (E.E.)

**Keywords:** ischemia reperfusion, liver regeneration, chemokines, neutrophils, oxidative stress, augmenter of liver regeneration

## Abstract

Hepatic ischemia reperfusion injury (IRI) is a major complication in liver resection and transplantation. Here, we analyzed the impact of recombinant human augmenter of liver regeneration (rALR), an anti-oxidative and anti-apoptotic protein, on the deleterious process induced by ischemia reperfusion (IR). Application of rALR reduced tissue damage (necrosis), levels of lipid peroxidation (oxidative stress) and expression of anti-oxidative genes in a mouse IRI model. Damage associated molecule pattern (DAMP) and inflammatory cytokines such as HMGB1 and TNFα, were not affected by rALR. Furthermore, we evaluated infiltration of inflammatory cells into liver tissue after IRI and found no change in CD3 or γδTCR positive cells, or expression of IL17/IFNγ by γδTCR cells. The quantity of Gr-1 positive cells (neutrophils), and therefore, myeloperoxidase activity, was lower in rALR-treated mice. Moreover, we found under hypoxic conditions attenuated ROS levels after ALR treatment in RAW264.7 cells and in primary mouse hepatocytes. Application of rALR also led to reduced expression of chemo-attractants like CXCL1, CXCL2 and CCl2 in hepatocytes. In addition, ALR expression was increased in IR mouse livers after 3 h and in biopsies from human liver transplants with minimal signs of tissue damage. Therefore, ALR attenuates IRI through reduced neutrophil tissue infiltration mediated by lower expression of key hepatic chemokines and reduction of ROS generation.

## 1. Introduction

Patients receiving an organ transplant face myriad potential complications, including tissue damage due to ischemia-reperfusion injury (IRI) and immunological rejection. In general, depending on the organ, IRI levels as low as 20% in an allograft can cause impaired function or even early organ failure [1]. Although it remains unclear how IRI affects the likelihood of organ rejection, it can be argued that it is a critical factor to the early immunological response that later provokes chronic transplant damage.

In the liver, the initial phase of ischemia reperfusion (IR) damage is characterized by enhanced levels of reactive oxygen species (ROS) which induce oxidative stress. ROS acts like cytotoxins, harming sinusoidal endothelial and parenchymal cells [2,3,4,5]. Furthermore, cytokines such as interleukin (IL)-1, tumor necrosis factor α (TNFα) and platelet-activating factor are released by activated Kupffer cells [2,4,5], leading to increased expression of adhesion molecules that attract leucocytes. In particular, polymorphonuclear granulocytes (PMN) migrate into the hepatocellular interstitial space, become activated, and then release oxygen radicals, proteases and hydrolytic enzymes [2,4,5]. Among the early T cell subpopulations reaching the graft, activated CD3^+^CD4^−^CD8^−^γδTCR^+^ innate effector cells (an unconventional T cell population) produce IL-17 and thereby further enhance inflammation and tissue damage [6]. In addition to the accumulation of leukocytes, increased adhesion of thrombocytes to endothelial cells leads to formation of microthrombi via leukocyte/platelet aggregation. This event adds to the initial inflammation by restricting blood perfusion, leading to the formation of ischemic infarct zones within the liver parenchyma [7].

The liver is known for its extraordinary regeneration potential and possesses several protective mechanisms that can counteract IR damage. Previous studies have shown that the endogenous hepatotrophic growth factor augmenter of liver regeneration (ALR; gene: *GFER*) supports liver regeneration and protects cells from injury [8]. ALR is constitutively expressed by the hepatic parenchymal cells (hepatocytes and cholangiocytes) and is located in mitochondria and the cytosol [8]. Human hepatocytes have mainly three isoforms of ALR, two long isoforms (21 and 23 kDa) located in the mitochondria and cytosol, and a short 15 kDa isoform, detected solely in the cytosol and released from cells [8,9]. An anti-apoptotic effect of the recombinant ALR (rALR, corresponds to 15 kDa isoform) has been demonstrated in different injury models in the liver. Application of rALR has been shown to reduce caspase 3/7 activity after free fatty acid-induced injury, accompanied by reduced bax expression [9], to diminish ethanol- and death ligand-induced apoptosis [10]. Furthermore, rALR lowers cell damage after partial hepatectomy by inducing expression of anti-oxidative clusterin and anti-apoptotic Bcl2, suggesting that rALR supports liver regeneration through attenuating apoptosis rather than inducing proliferation [11]. In addition, rALR reduces H_2_O_2_ -induced oxidative stress in human glioma [12] and neuroblastoma cells [13], while decreasing ROS and cytochrome c-related apoptosis. rALR promotes tissue regeneration, attenuates acute injury and improves cell survival after hepatocyte transplantation [14], as demonstrated by reduced allograft rejection and improved animal survival after liver transplantation in rats [15]. Consistent with the experimental studies mentioned above, this effect is likely mediated by lowering the inflammatory response and reducing pro-inflammatory cytokine production [14,15]. Furthermore, recombinant human ALR has been shown to reduce IRI in rat kidneys [16] and livers [17], accompanied by lower serum transaminases, diminished apoptosis and less infiltration of inflammatory cells (neutrophils), but details on how rALR exerts this beneficial effect on hepatic IRI are still missing. 

The aim of the present study was to analyze in vivo whether rALR application protects against hepatic IRI and, if so, begin to examine the mechanisms involved. For this purpose we used an IR mouse model analyzing the impact of rALR on hepatic tissue damage, apoptosis, oxidative stress and ROS generation by different cell types; recruitment and activation of IR-associated inflammatory cells was also examined. In addition, to put our main findings in the mouse model into a translational perspective, we studied key features of donor organ biopsies taken before and after liver reperfusion in liver transplant patients.

## 2. Materials and Methods

### 2.1. Mouse Liver IRI Model

**Figure 1:**
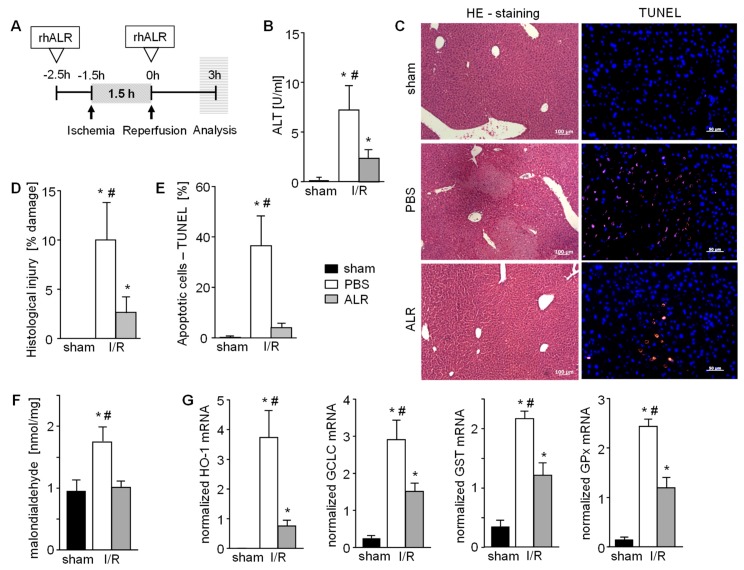
Augmenter of liver regeneration (ALR) reduces hepatic cell damage in a mouse model of ischemia reperfusion injury (IRI). (**A**) Schematic illustration of applied ischemia reperfusion (IR) mouse model. Mice were treated with ALR (100 µg/kg b.w.) at 2.5 h before ischemia (1.5 h) and beginning of reperfusion (3 h) (*n* = 6). (**B**) IR induced liver damage after 3h reperfusion was analyzed by quantification of ALT serum levels, histological examination of (**C**,**D**) necrotic areas performing H/E staining and (**C**,**E**) determination of apoptotic cells performing TUNEL assay (*n* = 6). (**F**) Oxidative stress as a marker of IRI in liver tissues after 3h reperfusion was analyzed by quantification of malondialdehyde (product of lipid peroxidation, normalized to sham) and (**G**) mRNA expression of anti-oxidative genes (HO-1, GCLC, GST, and GPX) reflecting cellular response to reactive oxygen species (ROS) (*n* = 5). Gene expression was normalized to 18S. * *p* < 0.05 or ^#^ *p* < 0.05 differs from sham or IR/ALR, respectively.

Experiments in mice were conducted according to the regulations of the State of Bavaria (permission granted by Regierung der Oberpfalz, 54-2532.1-2/12). Male 6–8 week old wild-type (wt) C57BL/6 (B6) mice were used (Charles River Laboratories, Sulzfeld, Germany), weighing 19–22 g and housed in standard polycarbonate mouse cages for at least ten days before experiments were started. All mice were kept under standard laboratory conditions (12 h light/dark cycle, 22 ± 2 °C; 60 ± 5% humidity). Partial liver IRI was induced according to Abe et al. [18] and as previously described [6]. Briefly, an atraumatic clip was placed across the portal vein, hepatic artery and bile duct just above branching to the right lateral lobe. This results in depriving 70% of the liver of blood flow. After 90 min of ischemia, the clamp was removed and the liver was reperfused. Blood and tissues were collected from anesthetized animals at specified times (3, 6 or 24 h). Sham-operated animals underwent the same procedure without clamping. Animals received i.p. injections of rALR (100µg/kg b.w. in 200 μL PBS) or PBS 60 min before ischemia and immediately before reperfusion (Figure 1A). The severity of ischemic injury was evaluated by measuring serum alanine amino transferase (ALT).

Recombinant human short form (15 kDa) ALR (rALR) was prepared as described previously [19], with some modifications. Briefly, non-conserved cysteines C74 and C85 in human ALR may account for oligomerization and therefore were mutated to Ala (C74A/C85A), as described previously [20]. Mutants showed the same behavior as wild-type short-form ALR [20].

### 2.2. Human Liver Biopsies

The study was conducted in accordance with the Declaration of Helsinki and the protocol was approved by the local ethical committee of the University of Regensburg (ethics statement IRI-P# 11-101-0163, University of Regensburg, Regensburg, Germany). Written informed consent forms were obtained from all participants. Biopsies from transplanted human livers were performed intraoperatively at the end of the back-table preparation (=pre-reperfusion) and before abdominal closure (=1.5 h post-reperfusion). An additional biopsy was taken if a so-called second look operation was necessary within 24–48h (=24–48 h post-reperfusion). Half of each liver tissue biopsy was immediately fixed in formalin and used for routine histological examination. A pathologist categorized these liver biopsy samples as “damage” or “no damage”. The second part of the biopsy core was stored in RNAlater^®^ for qRT-PCR analyses. All core biopsies had a length of at least 1.5 cm, a diameter from 1.2 to 1.8 mm, and in each case, more than 10 portal fields per biopsy could be found (for patient characteristics see Table S1).

### 2.3. Histological Analysis (Hematoxylin-Eosin)

Murine liver tissues 3 h post-reperfusion were harvest and embedded in paraffin for histological analysis. Sections measuring 4 µm were cut and stained with hematoxylin and eosin dye (H&E staining). Liver damage (percent necrosis) was determined morphometrically using a Zeiss AxioVision Module, where the percent necrosis was calculated from the total square micrometers of the tissue section; five sections from the ischemic part of the liver of each animal were measured (*n* = 8 animals/experimental point) [6].

### 2.4. TUNEL Assay—Analysis of Apoptosis

Apoptotic cells in liver tissue were quantified 3 h post-reperfusion using the TUNEL apoptosis detection assay from Millipore (Billerica, MA, USA), according to the manufacturer instructions. Nuclear staining was performed with propidium iodide (PI). Photomicrographs were taken using a Leica DM 4500B microscope and Leica DFC 290 digital camera system (Leica Microsystems, Wetzlar, Germany). Quantitative analysis was performed by counting positive nuclei [21].

### 2.5. Immunohistochemistry

Gr-1^+^ and CD3^+^ cells in mice were immunohistochemically stained on acetone-fixed frozen sections as previously described [6]. Briefly, dried sections were blocked with 10% goat serum (1 h), incubated with antibodies against Gr-1 and CD3 (1/100) for 30 min and with anti IgG-Alexa 594 Ab (1/200) plus DAPI (1/10,000) (more details see Table S2). Gr-1^+^ and CD3^+^ cells were counted in necrotic areas (NA) per high-power field (HPF) (×200 magnification; five HPF per slide, eight animals per group) and quantified by a blinded observer. Antibodies used in the study are listed in supplementary Table S2.

### 2.6. Isolation of Cells

For isolation of liver γδT cells, whole B6 livers were dissociated using the gentleMACS Dissociator (Miltenyi Biotec, Bergisch-Gladbach, Germany) and CD3^+^ γδTCR^+^ T cells were isolated using a presorting step with CD3^+^ immunomagnetic beads (Miltenyi Biotec) and then sorted by FACS (FACSAria; BD Biosciences, Heidelberg, Germany) while gating on γδTCR^+^ cells.

For isolation of Gr-1 cells, spleens were dissociated using the gentleMACS Dissociator (Miltenyi Biotec, Bergisch-Gladbach, Germany) and Gr-1^+^ cells were isolated using immunomagnetic beads (Miltenyi Biotec).

To obtain single cell suspensions of hepatocytes, livers were treated by a four-step perfusion protocol, as we and others have described previously [22]. The resulting cell suspension was separated by gradient centrifugation with 35% isotonic Percoll (Amersham Biosciences, Piscataway, NJ, USA) at 700× *g* for 20 min at 20 °C to select for viable single cells.

### 2.7. In Vitro Culture Conditions

After seeding 1 × 10^6^ purified γδT cells for 2 h in MEM alpha with 10% FCS and 1% penicillin/streptomycin (Sigma, Taufkirchen, Germany), cells were activated with 2 ng/mL concanavalin A (ConA) and incubated with rALR (100 ng/mL) for 48 h. Non-treated cells served as a negative control and ConA-stimulated cells served as a positive control, respectively.

Primary hepatocytes were seeded onto collagen-coated Corning BioCoat (Fisher Scientific, Schwerte, Germany) culture dishes and maintained in culture, as described previously [23]. RAW264.7 cells (ATCC no. TIB-71), a mouse macrophage cell line, were cultured in RPMI medium 1640 GlutaMAX (Fisher Scientific, Schwerte, Germany) with 5% fetal calf serum (FCS) and 1% penicillin/streptomycin.

For experiments under hypoxic conditions, cells were maintained in a hypoxia chamber at 37 °C, 5% CO_2_ and 1% O_2_ for the indicated times and subsequently maintained under normal culture conditions for reoxygenation.

### 2.8. Flow Cytometry and Cytokine Measurement

For flow cytometric analysis, cells were washed with phosphate-buffered saline (PBS) and resuspended in PBS. Cells were stained with antibodies for surface antigens (γδTCR, CD3) and for intracellular proteins (IFN-γ, IL-17A) listed in supplementary Table S2. Flow cytometry was performed on a FACSCanto II (BD Biosciences). Data were analyzed using FlowJo software (Tree Star, Ashland, OR). Cells from all displayed plots originate from positive gating on leukocytes and living cells, while excluding doublets.

Commercially available ELISA kits were used for determination of IL-17A (R&D Systems, Wiesbaden, Germany) and TNF-α (BD Biosciences, Darmstadt, Germany) levels in the culture supernatants.

### 2.9. Reactive Oxygen Substances and Oxidative Stress

The malondialdehyde (MDA) assay (BioVision, Milpitas, USA) was used to quantify lipid peroxidation resulting from oxidative stress and was performed according to the manufacturer’s protocol. Myeloperoxidase (MPO) is rapidly released by activated polymorphonuclear neutrophils and therefore MPO was analyzed to determine numbers of neutrophils as well as the potential appearance of hypochlorous acid (HOCl), which is a potent oxidant enzymatically produced by MPO during phagocytic lysis. MPO was analyzed with a quantification kit (Hycult Biotech, Uden, The Netherlands), according to the manufacturer’s protocol. Generation of superoxide anion by NADPH oxidase was analyzed by the NBT (nitrobluetetrazolium) assay, as described previously [24]. Production of cellular ROS was quantified by the CM-H_2_DCDFA assay. CM-H_2_DCFDA (5-(and-6)-chloromethyl-2′,7′-dichlorodihydrofluores cein diacetate) passively diffuses into cells, where its acetate groups are cleaved by intracellular esterases and its thiol-reactive chloromethyl group reacts with intracellular glutathione and other thiols. Subsequent oxidation yields a fluorescent adduct that is trapped inside the cell. A fresh stock solution of CM-H2DCFDA (0.87 mM; Life Technologies, Eugene, OR, USA) was prepared in DMSO and diluted to a final concentration of 10 μM in PBS. Cells were washed with PBS followed by incubation with 100 μL of working solution of CM-H_2_DCFDA for 30 min at 37 °C in the dark. Cell culture supernatant was removed, cells were washed with PBS, followed by measurement in a fluorescent plate reader (Excitation at 495 nm, emission at 530 nm). Relative fluorescence units were normalized to mg protein. 

### 2.10. Gene Expression (Quantitative Real-Time PCR)

Total RNA was isolated from mouse liver tissue using the RNeasy kit (Qiagen, Hilden, Germany). Subsequently, first strand cDNA was synthesized using 1 µg of total RNA and virus-reverse transcription reaction (QuantiTect reverse transcription kit, Qiagen, Hilden, Germany). Real-time PCR was performed in triplicates using the LightCycler^®^ SYBR Green 480 Master (Roche, Penzberg, Germany) and LightCyler^®^ 480 System (Roche, Penzberg, Germany). Quantification of transcript levels was performed using a standard curve of at least six different dilutions. The PCR reaction was evaluated by melting curve analysis and primers used for amplification are listed in supplementary Table S3. 

### 2.11. Western Blot Analysis

Murine liver samples or harvested cells from cell culture experiments were homogenized in 1 mL RIPA (150 mM NaCl, 1.0% Triton-X-100, 0.1% SDS, 50 mM Tris, pH 7.2) buffer using the gentleMACS dissociator (Miltenyi Biotec, Bergisch-Gladbach, Germany). Lysates were collected after centrifugation (4000× *g* for 5 min). ALR protein expression was determined by western blotting, as previously described [25]. Briefly, total protein homogenates (25 μg/lane) were separated by 12% SDS-PAGE under reducing conditions using ß-mercaptoethanol. Proteins were transferred onto polyvinylidene fluoride membranes and incubated overnight with anti-ALR AB (1:440) and anti-β-actin AB (1:1000). Western blot analysis for CXCL1, CXCL2, CCL2 and β-tubulin (for details see supplementary Table S2) were performed according to standard protocols and all western blots were developed with ECL reactions (Pierce, Rockford, IL, USA). 

### 2.12. Statistics

All data are presented as the mean ± standard deviation of the mean. Data were compared between groups using a Mann–Whitney test. A *p*-value of <0.05 was considered significant.

## 3. Results

### 3.1. Treatment with ALR Prevents Hepatic Ischemic Reperfusion Injury While Reducing Tissue Damage and Oxidative Stress

Hepatic IRI was induced in an in vivo mouse model, as depicted in Figure 1A, with or without application of recombinant human ALR at indicated times. Liver tissue of mice treated with PBS demonstrated 3 h post reperfusion greater serum ALT levels (Figure 1B), cell and tissue damage (necrosis) (Figure 1C,D), as well as an increased number of TUNEL positive cells in the hepatic parenchyma (Figure 1C,E), compared to sham animals. In comparison, ALR treatment reduced hepatic IRI, as shown by a clear reduction of ALT levels (Figure 1B), tissue damage (Figure 1C,D) and TUNEL positive cells (Figure 1C,E). Enhanced ROS, mainly responsible for IR-related tissue damage, is shown by increased MDA levels that reflect enhanced lipid peroxidation (Figure 1F). Furthermore, anti-oxidative gene expression, like HO-1 (heme oxidase-1), GCLC (glutamate cysteine ligase catalytic subunit), GST (glutathione S-transferase) and GPx (glutathione peroxidase), were increased after IR (Figure 1G). ALR treatment prevented an increase in MDA levels (Figure 1F), and significantly reduced expression of the ROS-sensitive genes (Figure 1G), indicating less oxidative stress in tissues treated with ALR. Therefore, hepatic IRI is significantly diminished by ALR treatment, leading to reduced ROS levels and less tissue damage.

### 3.2. Recruitment and Activation of γδT Cells (CD3^+^) Remains Unaffected by ALR Treatment during IR

**Figure 2:**
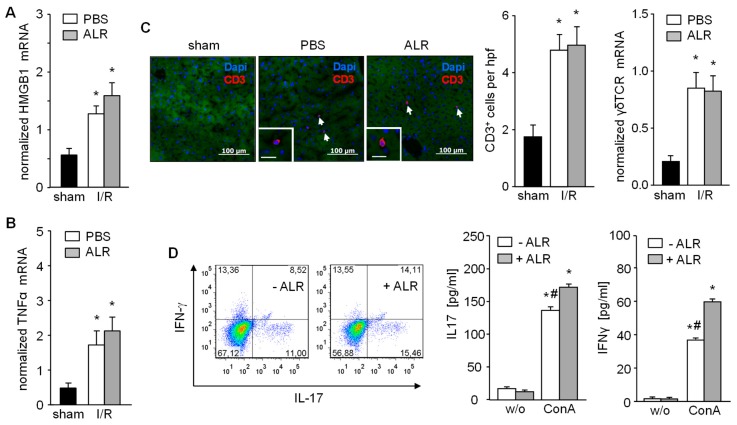
Expression of HMGB1 and TNFα, recruitment of CD3^+^ and activation of γδT cells after IR are not diminished upon ALR treatment. Liver tissues from IR mice were analyzed for (**A**) HMGB1 and (**B**) TNFα mRNA expression (*n* = 5). (**C**) Liver tissues were immuno-stained for CD3^+^ cells and number of infiltrated CD3^+^ cells were analyzed in each group (*n* = 6). Expression of γδT cell receptor (γδTCR) mRNA in liver tissue samples was analyzed by qRT-PCR (*n* = 5). Gene expression was normalized to 18S. * *p* < 0.05 differs from sham. (**D**) γδT cells were isolated from mouse livers and treated in culture with or without rALR (100 ng/mL, 48 h) followed FACS analysis for IL-17 and IFN-γ expression (*n* = 3). Furthermore, release of IL-17 and IFN-γ into the supernatant of cultured γδT cells after stimulation with concanavalin A (ConA, 2 ng/mL, 48 h) in the presence or absence of rALR (100 ng/mL, 48 h) were determined by ELISA (*n* = 3). * *p* < 0.05 or ^#^ *p* < 0.05 differs from unstimulated or corresponding stimulated γδT cells, respectively.

In the early phase of IRI, hepatocytes release DAMP molecules, including HMGB1, which activate Kupffer cells for further release of TNFα. ALR treatment did not alter either HMGB1 (Figure 2A) or TNFα mRNA expression in liver tissue from IR mice, compared to the PBS control group after 3 h of reperfusion (Figure 2B). The inflammatory response plays a critical role in hepatic IRI, including the infiltration of immune cells (e.g., T cells) which are key mediators of IRI pathogenesis [2,6]. Therefore, liver tissue sections were immuno-stained for CD3^+^ cells (T cells) and, indeed, increased numbers of infiltrating CD3^+^ cells were found in both PBS- and ALR-treated mice, compared to sham mice (Figure 2C). In addition, γδT cells, a subpopulation of IL-17-secreting CD3^+^ cells, was also not affected in number by ALR treatment, since γδT receptor (γδTCR) mRNA expression in IR liver tissue appeared relatively unaltered compared to PBC controls (Figure 2C). In addition, γδTCR positive cells isolated from mouse liver tissue were stimulated in vitro with lymphocyte-activating ConA in the absence or presence of ALR (Figure 2D). Flow cytometry and ELISA analysis of cultured γδTCR^+^ cells and their supernatants revealed that ConA caused a potent stimulation of IL17 and IFN-γ production, which was further enhanced with ALR treatment (Figure 2D). Therefore, our results indicate that ALR treatment during IR does not alter release of DAMPs (i.e., HMGB1) from hepatocytes or TNFα from Kupffer cells, and has little appreciable effect on γδT/CD3^+^ cells infiltrating the liver.

### 3.3. ALR Attenuates Hepatic Infiltration of Neutrophils (Gr1^+^ ) and Reduces ROS Generation by Macrophages (RAW 264.7) and Hepatocytes

**Figure 3:**
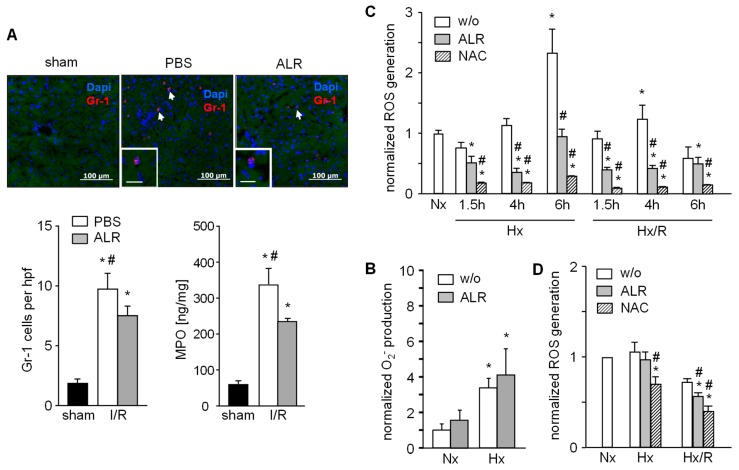
ALR decreases number of infiltrating neutrophils (GR-1) in an IRI mouse model and reduces generation of ROS in macrophages (RAW264.7) and primary hepatocytes upon hypoxia/reoxygenation. (**A**) Liver tissues from IR mice were immuno-stained for GR-1 and the number of infiltrated GR-1 positive cells were analyzed in each group. In addition, neutrophil resident myeloperoxidase (MPO) in these samples was analyzed (*n* = 6). * *p* < 0.05 or ^#^ *p* < 0.05 differs from sham or IR/ALR group, respectively. (**B**) Gr-1 positive cells were isolated from mouse spleen and analyzed for O_2_^−^ release into culture medium after 1 h of normoxia (Nx) or hypoxia (Hx) without (w/o) or with ALR (100 ng/mL) treatment (*n* = 3). (**C**) RAW 264.7 cells (mouse macrophage cell line) were subjected to hypoxia or hypoxia with 2 h of reoxygenation (Hx/R) for the indicated times, in the absence or presence of rALR (100 ng/mL), following analysis of oxygen radical generation (*n* = 3). Treatment with radical scavenger 10 mM *n*-acetylcystein (NAC) was used as positive control. (**D**) Primary mouse hepatocytes were subjected for 4 h to hypoxia or hypoxia with 2 h of reoxygenation in the absence or presence of rALR (100 ng/mL) following analysis of oxygen radical generation by performing a H_2_DCFDA assay. Results (*n* = 4) are normalized to Nx with * *p* < 0.05 or ^#^ *p* < 0.05 differs from Nx or corresponding HX, Hx/R w/o ALR treatment, respectively.

Since the IR inflammatory response includes infiltration and activation of neutrophils, we analyzed GR1^+^ cells by immunostaining in mouse liver sections. Livers from mice with IRI showed an increased number of GR1^+^ cells, but ALR treatment significantly attenuated the infiltration of GR1^+^ cells compared to the PBS group (Figure 3A). In addition, neutrophil resident myeloperoxidase (MPO) was significantly lower in hepatic tissue from IR mice treated with ALR compared to PBS (Figure 3A). Neutrophils (GR1^+^ cells) isolated from mouse spleen were cultured under normal (normoxia, Nx) and hypoxic (hypoxia, Hx) conditions for 1 h and generation of superoxidanion (O_2_^−^) was analyzed. Increased levels of O_2_^-^ were seen after Hx with control (without ALR) and ALR-treated cells (Figure 3B). RAW264.7 cells (a mouse macrophage cell line) were cultured for indicated times under Nx, Hx and Hx with an additional 2 h of reoxygenation (Hx/R), followed by analysis of ROS generation (Figure 3C). ALR treatment significantly reduced ROS levels in mouse macrophages (RAW264.7) under Hx and Hx/R conditions (Figure 3C). Furthermore, ROS generation in RAW264.7 cells after addition of lipopolysaccharide was significantly reduced by ALR treatment (Figure S1). Additional, primary mouse hepatocytes were cultured under conditions for Nx, Hx or Hx/R and ALR treatment slightly, but significantly, diminished ROS release after Hx/R in hepatocytes (Figure 3D). Taken together, these results demonstrate that ALR reduces oxidative stress in IRI in-part by reducing ROS generation in macrophages (Kupffer cells) and hepatocytes, and by lowering the number of infiltrating—MPO expressing—GR1^+^ cells in IR liver tissue.

### 3.4. Expression of Chemo-Attractants in IRI Liver Tissue and Primary Hepatocytes Cells is Mitigated by ALR

**Figure 4:**
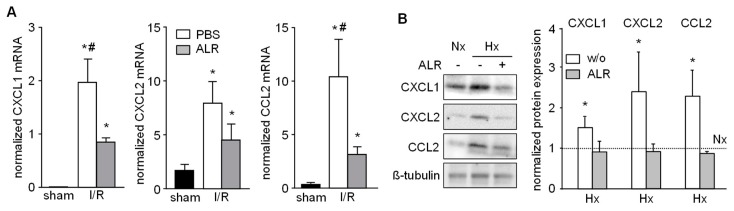
ALR attenuates hepatic chemokine expression in an IRI mouse model and in primary hepatocytes. (**A**) Liver tissues from IRI mice were analyzed for mRNA expression of chemo-attractants CXCL1 (Gro-α, KC), CXCL2 (Gro-β, MIP-2) and CCL2 (MCP-1) by qRT-PCR (*n* = 6; gene expression was normalized to 18S). * *p* < 0.05 or ^#^ *p* < 0.05 differs from sham or IR/ALR group, respectively. (**B**) Primary mouse hepatocytes were subjected to Nx or Hx in absence or presence of rALR (100 ng/mL). Protein expression of CXCL1, CXCL2 and CCL2 was analyzed by western blotting. Immunoblots from three different experiments were analyzed by densitometric analysis, normalized to Nx (dotted line); one representative is shown (for all data see Figure S3). * *p* < 0.05 differs from Nx.

Chemokines known to attract inflammatory cells, that is, neutrophils, infiltrating into injured liver tissue were analyzed in our IRI model. Hepatic tissue from IR mice revealed enhanced mRNA expression of CXCL1 (Gro-α, KC), CXCL2 (Gro-β, MIP-2) and CCL2 (MCP-1), compared to sham-operated mice (Figure 4A). ALR treatment significantly reduced the extent of CXCL1 and CCL2 mRNA expression in IRI mice, compared to PBS group (Figure 4A). Furthermore, primary mouse hepatocytes were cultured under conditions for Nx or Hx and analyzed for chemokine expression by western blotting (Figure 4B and Figure S3). Hepatocytes treated with rALR showed diminished expression of CXCL1, CXCL2 and CCl2 under conditions of Hx, compared to without ALR incubation. ALR treatment of RAW264.7 cells did not result in altered mRNA expression of these same chemokines (data not shown). Overall, these results suggest that less neutrophil infiltration in IR mouse livers might be due to reduced expression of chemoattractants in, for example, hepatocytes after ALR treatment.

### 3.5. Expression of ALR is Increased in an IR Mouse Model and in Human Trans-planted Liver Tissue without Injury after Reperfusion

**Figure 5:**
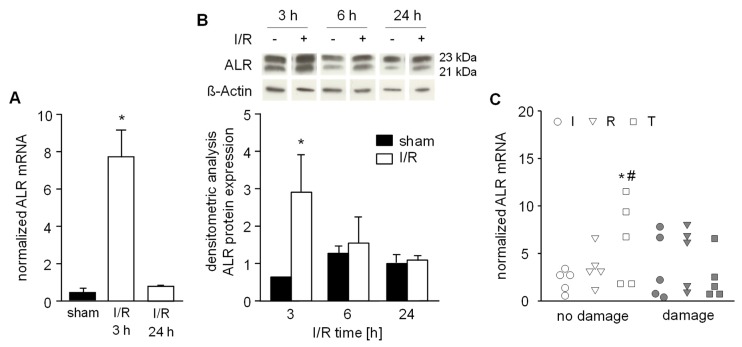
Hepatic ALR expression after ischemic reperfusion. A) Mice were subjected to ischemia as described in Material and Methods and liver tissue samples were taken after 3 h or 24 h (*n* = 6 each) of reperfusion. (**A**) ALR mRNA and (**B**) ALR protein expression in liver tissue samples were analyzed by qRT-PCR and western blotting, respectively. Expression of ALR mRNA was normalized to 18S, ALR protein to ß-actin and sham operated animals (*n* = 3) served as control. * *p* < 0.05 differs from sham. Immunoblots from 3 different experiments were analyzed by densitometric analysis and one representative blot is shown (for all data see Figure S4). **C**) Human liver biopsies were obtained from livers prior to transplantation (I: after ischemia, pre-reperfusion, *n* = 10), immediately before abdominal closure (R: approximately 0.5 h post-reperfusion, *n* = 10) and during a 2nd look operation (T: 24 h–48 h after reperfusion, *n* = 10). Liver biopsy samples were examined by a pathologist evaluating HE histochemistry and assigned according to “no damage” (<5% necrosis) or observed tissue “damage” (>5% necrosis). Expression of ALR mRNA was normalized to YWHAZ with * *p* < 0.05 or ^#^ *p* < 0.05 differs from ischemia with no damage or reperfusion with damage, respectively.

Anti-apoptotic and anti-oxidative properties are ascribed to ALR and its expression is regulated among others by Nrf2 (nuclear factor erythroid 2-related factor 2), which is known as a ROS sensitive transcription factor [25]. Therefore, we analyzed liver tissue samples from IR mice after 3, 6 and 24 h of reperfusion for ALR expression. We found a distinct induction of ALR mRNA and protein expression after 3 h in the early phase of reperfusion and no difference in expression after 6 or 24 h reperfusion, compared to sham-operated mice (Figure 5A,B, Figure S4). Notably, the anti-oxidative genes HO-1, GCLC, GST and GPx were upregulated within 3 h of IR (see Figure 1G); these genes are known to be regulated by Nrf2 [25].

Furthermore, we analyzed human liver tissues biopsies for ALR mRNA expression taken at the end of the back-table preparation (“I”: after cold ischemia, but before organ reperfusion) and immediately before abdominal closure (“R”: approximately 0.5 h post-reperfusion). In addition, at 24–48 h posttransplantation these patients underwent a second look operation where a third biopsy was taken (“T”: 24–48 h after reperfusion). Liver biopsies were analyzed for IR-related tissue damage by histochemistry and for ALR mRNA expression by RT-PCR. Interestingly, we found that patients who did not develop IR-related damage after reperfusion demonstrated significantly increased ALR expression compared to ischemia within the “no damage” group and to corresponding patients with damage after reperfusion (Figure 5C).

## 4. Discussion

**Figure 6:**
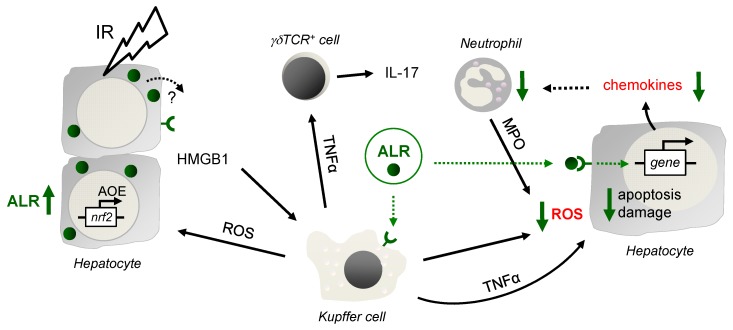
Schematic illustration of the findings. After IR, in the early phase of liver injury, activated Kupffer cells (KC) release reactive oxygen species (ROS), which stress and harm hepatocytes, leading to induction of anti-oxidative gene expression such as genes regulated by ROS sensitive transcription factor Nrf2. Nrf2 is known to regulate expression of genes harboring an anti-oxidative response element (ARE) in their promotor region, such as HO-1, GCLC, GPx and GST, but also ALR. In addition, hepatocytes release DAMPs (damage-associated molecular patterns), for example, HMGB1, which bind to Toll like receptor 4 on, for example, Kupffer and dentritic cells to propagate the inflammatory response, including TNFα expression and release. After IR, CD3^+^ cells are recruited to the liver, of which a subset are γδT cells, activated by TNFα, among others, and upregulate and release IL-17 and IFN-γ. Application of ALR does not alter HMGB1 and TNFα expression in IR-injured mouse livers, although ALR receptors are described on hepatocytes and Kupffer cells. A potential impact of ALR on γδT cell activity is not seen, since the expression and release of IL-17 and IFN-γ is almost unchanged. ALR reduces chemokine expression in IR injured livers and hepatocytes may be the responsible cell type. Therefore, the beneficial effect of ALR on IRI is most likely due to less oxidative stress by mitigating ROS generation in Kupffer cells and hepatocytes, reducing recruitment, but not activity of neutrophils (deliver ROS by MPO), which results in attenuated hepatocyte death and tissue damage.

Ischemia reperfusion injury is a major complication occurring with vascular occlusion during liver surgery or during liver transplantation. The pathophysiology of hepatic IRI encompasses mainly oxidant stress and inflammatory responses contributing to various degrees of liver damage [26]. Current research addresses a better understanding of the cellular and molecular mechanisms of the hepatic IRI, which may lead to new concepts for therapeutic modalities. While liver repair and regeneration after IRI are critically important, their regulating mechanisms are not fully elucidated. Therefore, we analyzed ALR, an anti-oxidative and anti-apoptotic protein with beneficial effects on liver regeneration, in an IRI mouse model. In this study we show that ALR treatment results in lower oxidative stress, apoptosis, and diminished expression of chemoattractants, resulting in less infiltration of Gr-1 cells and reduced hepatic tissue damage after IRI (Summary in Figure 6).

Hepatocyte cell death during IRI occurs in a more-or-less hypoxic environment and the early phase of liver injury is characterized by activation of Kupffer-cells (KC), which release ROS and proinflammatory cytokines [4,5]. More specifically, ROS formation by KC is triggered by ischemic stress and by DAMPs such as HMGB1, which are released from hepatocytes during reperfusion [4,27]. While ALR treatment did not change levels of HMGB1 in hepatic tissue from IRI mice, ALR treatment reduced ROS formation in RAW264.7 cells suggesting a direct effect of ALR on ROS generation by KC in IRI livers. The source of hepatic ROS accumulation during IRI has been attributed to different cell types such as KC, but also neutrophils and hepatocytes [4,28]. Similar to KC, ROS from neutrophils are generated outside the cell and can diffuse into hepatocytes [3,29]. In our study, ALR could not reduce superoxide anion generation in neutrophils under hypoxic conditions and, therefore, lower hepatic MPO levels in IR mice after ALR treatment may be explained by fewer infiltrating neutrophils. In addition, we have shown that ALR can reduce ROS generation in primary hepatocytes, which may be due to reduced CYP2E1 activity after ALR treatment [30,31]. Less is known about how ALR performs its anti-oxidative effect, but the direct effect of ALR on KC and hepatocytes might be related to binding of ALR to its receptor on both KC and hepatocytes subsequently activating NFκB expression [8]. NFκB target genes were shown to attenuate ROS promoting cell survival [32]. A signaling pathway mainly involved in this scenario is the crosstalk of NFκB with JNK, preventing cell death through apoptosis and necrosis [32]. In summary, ALR treatment reduces hepatic oxidative stress under IR conditions, which is not mediated by altered MPO activity of neutrophils, but via decreased ROS generation by KC and hepatocytes.

The late phase of IRI is characterized by enhanced expression of chemotactic agents (chemokines), cell adhesion molecules and consequently recruitment of effector cells such as neutrophils [4,5]. It was reported previously that ALR treatment attenuates leukocyte (neutrophil) migration as well as recruitment of infused splenic CD4^+^ T cells in IR livers [17]. In the liver, IL-17 is mainly released after IR from CD3^+^ γδTCR^+^ cells [6] and to a minor degree from conventional CD4^+^ Th17 cells [33], and facilitates the recruitment and migration of neutrophils. In our study, we confirmed that ALR treatment reduced neutrophils in liver tissue of IRI mice and, furthermore, demonstrated that this attenuated infiltration of neutrophils is independent of IL-17 release, since γδTCR^+^ cells are unchanged in number or activity. However, we found that ALR treatment reduces chemokine expression in IR mouse liver tissue (CXCL1, CCL2) and hypoxic primary hepatocytes (CXCL1, CXCL2, CCL2), suggesting an attenuated chemotaxis and therefore less migration of neutrophils in hepatic injury [34]. Interestingly, tissue levels of TNFα, an important inducer of hepatocytes and KCs to produce neutrophil chemoattractants [35], were not changed after ALR treatment in IRI mice, even though ALR was reported to induce secretion of TNFα and IL6 from rat KC [36]. Otherwise, activated KCs generate ROS, which in turn induce expression and release of CXCL1 and CXCL2 in hepatocytes [4,5,37]. Additionally, injured hepatocytes or KCs are thought to be the primary source of hepatic CCL2 [38]. Moreover, lipid peroxidation products were described as chemotactic factors, thereby amplifying the inflammatory response during reperfusion [39]. Therefore, by reducing ROS and subsequently lipid peroxidation products and cell damage, ALR treatment attenuates hepatic chemokine expression and neutrophil recruitment in IRI livers. 

In addition, we suppose that ALR may directly regulate chemokine expression, since rALR treatment of primary human hepatocytes reduced expression of various chemokines (supplementary Figure S2), which were shown to be important in fibrosis and liver inflammation [40]. Particularly, the expression of CXCL1, CXCL5 and CXCL6, which are chemoattractants for neutrophils, is attenuated after ALR treatment. In hepatocytes, the expression of chemokines such as CXCL1, CXCL2 and CCL2 is primarily controlled by NFκB and activator protein 1 (AP-1) [41,42,43]. Furthermore, it has been shown that ALR treatment of hepatocytes activates PI3K/Akt and MAPK signaling pathways, leading to increased activity of NFκB and AP-1, respectively [8]. In addition, Stat3 in hepatocytes is involved in induction of CXCL2 and CCL2 expression followed by increased infiltration of inflammatory cells in an alcohol liver injury model [44]. We have reported earlier that ALR modulates the IL6 signaling pathway by reducing the activation of STAT3 and subsequent expression of acute phase protein [19]. Therefore, ALR likely regulates chemokine expression in addition to reduced ROS levels by activating PI3K/Akt and MAPK signaling pathways leading reduced Stat3 and enhanced NFκB and AP-1 activation. 

ALR is expressed in hepatic tissue from patients with liver diseases at various levels (summarized in [8]). ALR expression was found to be strongly increased in tissues with normal or abnormal regeneration activity like cirrhosis or hepatocellular carcinoma. We show in this study that hepatic ALR expression is increased in an IRI mouse model and in human liver tissue after liver transplantation. Enhanced ALR expression in human liver tissue after liver transplantation is associated with no tissue damage indicating successful liver regeneration or protection of oxidative stress and the inflammatory response. ALR expression in IRI livers is most likely induced by Nrf2, a transcription factor known to regulate ALR expression by binding to an anti-oxidant response element (ARE) [25]. Nrf2 acts as a master regulator of the antioxidant response during IR-induced oxidative stress and inflammation [18,26], while regulating expression of anti-oxidant genes, whose enzymes are involved in eliminating excessive ROS like GCLC, HO-1 and GST [45]. A previous report described a beneficial effect of over-expressing ALR (long from, 23 kDa) on oxidative stress and mitochondrial function in steatotic hepatocytes after IR [46]. Therefore, expression of ALR in transplanted liver tissue may be seen as part of the antioxidant response indicative for active regeneration, thereby preventing or reducing tissue damage. In addition, if ALR is released from hepatocytes it may act in a paracrine fashion through the above mentioned mechanisms.

In consideration of the fact that gene therapy for human diseases may not be at a developmental stage for therapeutic application, the production and use of recombinant proteins could have a significant impact on disease due to improvements in production of these biomolecules. Therefore, our results suggest that treatment with ALR during liver transplantation might improve outcomes through attenuating tissue damage after IR by anti-oxidative and anti-inflammatory mechanisms.

## Supplementary Materials

The following are available online at https://www.mdpi.com/2073-4409/8/11/1421/s1, Figure S1: ALR reduces generation of reactive oxygen species in macrophage cell line (RAW264.7) after lipopolysaccharide (LPS) treatment, Figure S2: ALR reduces chemokine mRNA expression in freshly isolated primary human hepatocytes (PHH), Figure S3: ALR attenuates hepatic chemokine expression in primary hepatocytes, Figure S4: Hepatic ALR expression after ischemic reperfusion, Table S1: Patient characteristics, Table S2: Antibodies used in the study, Table S3: Primers for RT-PCR.
