# Supplementary material for: Augmenter of Liver Regeneration Reduces Ischemia Reperfusion Injury by Less Chemokine Expression, Gr-1 Infiltration and Oxidative Stress
Source: Cells. 2019 Nov 12;8(11):1421. doi: 10.3390/cells8111421 (PMC6912457; doi:10.3390/cells8111421)
Supplement: Supplementary file 1 [file cells-08-01421-s001.pdf]

Reduced ischemia reperfusion injury after treatment with augmenter of liver regeneration by less chemokine expression, Gr-1 infiltration, oxidative stress and tissue damage

Thomas S. Weiss [1, 3*], Madeleine Lupke [1], Rania Dayoub[1], Edward K. Geissler [2], Hans J. Schlitt [2], Michael Melter [1], Elke Eggenhofer [2]

Supplementary Materials:

Human liver tissue for cell isolation was obtained from liver resections of patients undergoing partial hepatectomy for metastatic liver tumors of colorectal cancer. Primary human hepatocytes (PHH) were isolated and cultivated as described recently [1]. Briefly, non-neoplastic tissue samples from liver resections were obtained from patients undergoing partial hepatectomy for metastatic liver tumors of colorectal cancer. PHHs were isolated using a modified two-step ethylene glycol tetra-acetic acid (EGTA)/collagenase perfusion procedure and plated on collagen coated dishes. Experimental procedures were performed according to the guidelines of the charitable state controlled foundation HTCR (Human Tissue and Cell Research, Regensburg, Germany), with the written informed patient's consent. The study and the consent form were approved by the local ethical committee of the University of Regensburg (ethics statement 12-101-0048, University of Regensburg, Germany). All experiments involving human tissues and cells have been carried out in accordance to The Code of Ethics of the World Medical Association (Declaration of Helsinki).

Table S1: Patient characteristics.

Study groups		damage (n = 5)	no damage (n = 5)	*p*-value
Recipient				
Age (years ± SD)		49.8 ± 5.4	52.6 ± 75.0	0.714
Gender, n (%)	Male	3 (60)	2 (40)	0.579
	Female	2 (40)	3 (60)	
End stage of liver disease etiology, n (%)	Hepatitis	1 (20)	1 (20)	0.920
	Alcohol	3 (60)	4 (80)	
	Other	1 (20)	0 (0)	

Table S2: Antibodies used in the study.

Antibody	Cat. No.	Clonality/host	Supplier	Species
Primary antibodies				
CD3	47-0032	mono / rat	Invitrogen, Carlsbad CA, USA	m
Gr1	553127	mono / rat	BD Biosciences, Franklin Lakes NJ, USA	m
γδ-TCR	17-5711-82	mono / ah	Invitrogen, Carlsbad CA, USA	m
IFN-γ	11-7311-82	mono / rat	Invitrogen, Carlsbad CA, USA	m
IL-17	560522	mono / rat	BD Biosciences, Franklin Lakes NJ, USA	m
CXCL1	Ab86436	poly / rabbit	Abcam, Cambridge, UK	h, m, rt
CXCL2	Ab18949	poly / rabbit	Abcam, Cambridge, UK	m
CCL2	Ab9899	poly / rabbit	Abcam, Cambridge, UK	m
β-tubulin	Ab59680	poly / rabbit	Abcam, Cambridge, UK	h, m, rt
β-actin	4970	mono / rabbit	Cell Signaling, Danvars MA, USA	h, m, rt, mk, b, pg
Secondary antibodies				
Alexa 594 IgG	A-11007	poly / goat	Invitrogen, Carlsbad CA, USA	r
IgG-HRP	P0048	poly / goat	Dako, Hamburg, Germany	rb

Abbreviations used are: mono, monoclonal antibody; poly, polyclonal antibody; h, human; ah, Armenian hamster; m, mouse; rt, rat; rb, rabbit; g, goat; mk, monkey; b, bovine; pg, pig.

Table S3: Primers for RT-PCR.

Gene	Accession Nr.	Primer sequence (5′ - 3′)	
Mouse ALR	NM_023040	*Fwd.*	cac agg atc ggg aag aat tg
		Rev.	att cct cgc agg ggt aaa ac
Mouse HO-1	NM_010442	*Fwd.*	gtc aag cac agg gtg aca ga
		Rev.	tgt ctg tga ggg act ctg gtc
Mouse GCLC	NM_010295	*Fwd.*	aga tgc gga ggc atc aaa
		Rev.	tat gct gca ggc ttg gaa t
Mouse GST	NM_013541	*Fwd.*	cac cct cat cta cac caa cta tga
		Rev.	agc ttt gcc tcc ctg gtt
Mouse GPx	NM_008160	*Fwd.*	ttt ccc gtg caa tca gtt c
		Rev.	ttc tca cca ttc act tcg ca
Mouse HMGB1	NM_001313894	*Fwd.*	atg ggc aaa gga gat cct a
		Rev.	att cat cat cat cat ctt ct
Mouse TNFα	NM_013693	*Fwd.*	acg gca tgg atc tca aag ac
		Rev.	gtg ggt gag gag cac gta gt
Mouse γδTCR	NG_007033	*Fwd.*	ctgtgcctgaaagggcaatg
		Rev.	tagtaggcagaggtgctcgt
Mouse CXCL1	NM_008176	*Fwd.*	ctt gaa ggt gtt gcc ctc ag
		Rev.	tgg gga cac ctt tta gca tc
Mouse CXCL2	NM_009140	*Fwd.*	cgcccagacagaagtcatag
		Rev.	tcctcctttccaggtcagtta
Mouse CCL2	NM_011333	*Fwd.*	cat cca cgt gtt ggc tca
		Rev.	gct gct ggt gat cct ctt gta
Mouse CCL3	NM_011337	*Fwd.*	atg aag gtc tcc acc act gcc ctt g
		Rev.	ggc att cag ttc cag gtc agt gat
Mouse18S	X03205	*Fwd.*	gtaacccgttgaaccccatt
		Rev.	ccatccaatcggtagtagcg
Human ALR	NM_005262	*Fwd.*	gaa gcg gga cac caa gtt ta
		Rev.	ttc agc aca ctc ctc aca gg
Human YWHAZ	NM_003406	*Fwd.*	gca att act gag aga caa ctt gac a
		Rev.	tgg aag gcc ggt taa ttt t
Human CXCL1	NM_001511	*Fwd.*	aac ccc aag tta gtt caa tct gga
		Rev.	cat gtt gca ggc tcc tca gaa
Human CXCL5	NM_002994	*Fwd.*	cat cgc cag cgc tgg tcc t
		Rev.	ggg atg aac tcc ttg cgt ggt ct
Human CXCL6	NM_002993	*Fwd.*	gtt tac gcg tta cgc tga gag taa a

		Rev.	cgt tct tca ggg agg cta cca
Human CCL3	NM_002983	*Fwd.*	cag aat cat gca ggt ctc cac
		Rev.	gcg tgt cag cag caa gtg
Human CCL5	NM_002985	*Fwd.*	ggc agc cct cgc tgt cat cct ca
		Rev.	ctt gat gtg ggc acg ggg cag tg

Supplementary Figure

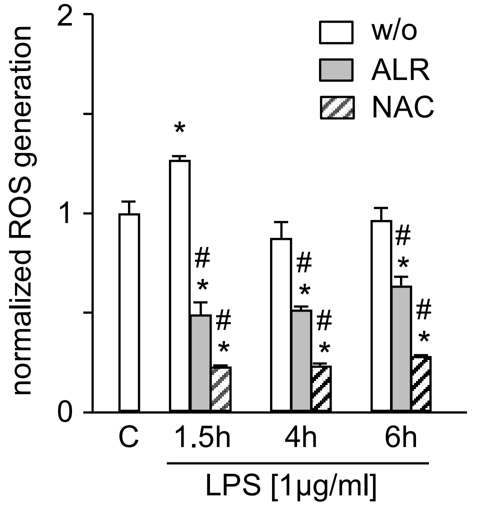

Figure S1: ALR reduces generation of reactive oxygen species in macrophage cell line (RAW264.7) after lipopolysaccharide (LPS) treatment. RAW 264.7 cells (mouse macrophage cell line) were treated with 1µg/ml LPS (known to induce ROS) for the indicated times, in absence or presence of rALR (100ng/ml), following analysis of oxygen radical generation. Treatment with radical scavenger 10 mM N-acetylcystein (NAC) was used as positive control. Results (n=4) are normalized to control (C), untreated cells. * $p < 0.05$ or # $p < 0.05$ differs from C or corresponding cells w/o ALR treatment, respectively.

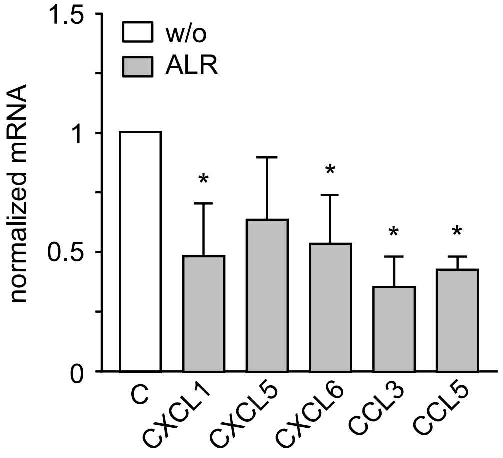

Figure S2: ALR reduces chemokine mRNA expression in freshly isolated primary human hepatocytes (PHH). PHHs were seeded and after 24 h cells were treated with 100ng/ml rALR for 24h followed by analysis of CXCL1 (Gro-α, KC), CXCL5 (ENA-78), CXCL6 (GCP-2), CCL3 (MIP-1α) and CCL5 (RANTES) mRNA expression performing qRT-PCR. Cell isolation results in stress-induced activation of hepatocytes towards regeneration and altered metabolism accompanied by higher susceptibility for ALR induction [2]. Gene expression was normalized to control, untreated cells (n=3). * $p < 0.05$ differs from control.

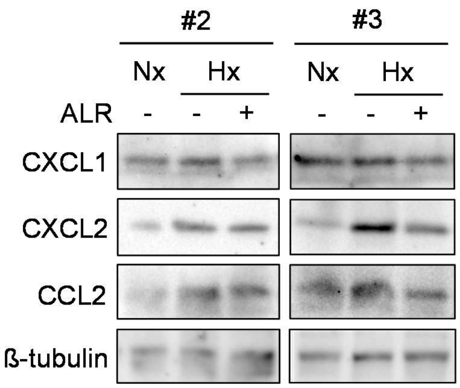

Figure S3: ALR attenuates hepatic chemokine expression in in primary hepatocytes. Primary mouse hepatocytes were subjected to Nx or Hx in absence or presence of rALR (100 ng/ml). Protein expression of CXCL1, CXCL2 and CCL2 was analyzed by western blotting. Immunoblots from additional two different experiments corresponding to Figure 4 B (#2, #3) are shown.

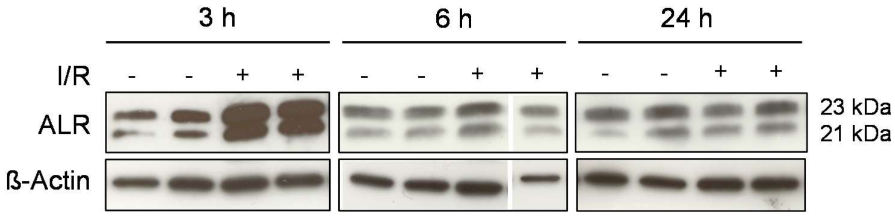

Figure S4: Hepatic ALR expression after ischemic reperfusion. Mice were subjected to ischemia as described in Material and Methods and liver tissue samples were taken after 3 h or 24 h of reperfusion. ALR protein expression in liver tissue samples were analyzed by western blotting. Immunoblots from additional two different experiments corresponding to Figure 5 are shown.

References:

1. Weiss, T.S.; Dayoub, R. Thy-1 (CD90)-Positive Hepatic Progenitor Cells, Hepatoctyes, and Non-parenchymal Liver Cells Isolated from Human Livers. *Methods Mol Biol* **2017**, *1506*, 75-89, doi:10.1007/978-1-4939-6506-9_5.
2. Thasler, W.E.; Dayoub, R.; Muhlbauer, M.; Hellerbrand, C.; Singer, T.; Grabe, A.; Jauch, K.W.; Schlitt, H.J.; Weiss, T.S. Repression of cytochrome P450 activity in human hepatocytes in vitro by a novel hepatotrophic factor, augmenter of liver regeneration. *J Pharmacol Exp Ther* **2006**, *316*, 822-829, doi:10.1124/jpet.105.094201.
